# Supplementary material for: The Diverse Binding Modes Explain the Nanomolar Levels of Inhibitory Activities Against 1-Deoxy-d-Xylulose 5-Phosphate Reductoisomerase from Plasmodium falciparum Exhibited by Reverse Hydroxamate Analogs of Fosmidomycin with Varying N-Substituents
Source: Molecules. 2024 Dec 28;30(1):72. doi: 10.3390/molecules30010072 (PMC11721986; doi:10.3390/molecules30010072)
Supplement: Supplementary file 1 [file molecules-30-00072-s001.zip › molecules-3370087-supplementary.pdf]

Supplementary Information

Table of contents

Figure S1. Stereo diagrams showing the |*F*o| - |*F*c| omit maps of the bound inhibitors in the active site of subunit B of *Pf*DXR.
Table S1. Data collection statistics for the ternary (enzyme - Mn^{2+} - inhibitor) and quaternary (enzyme - Mn^{2+} - NADPH - inhibitor) complexes of *Pf*DXR.
Table S2. Refinement statistics for the ternary (enzyme - Mn^{2+} - inhibitor) and quaternary (enzyme - Mn^{2+} - NADPH - inhibitor) complexes of *Pf*DXR.
Table S3. Summary of enzyme-inhibitor interactions observed in the crystal structures of *Pf*DXR.

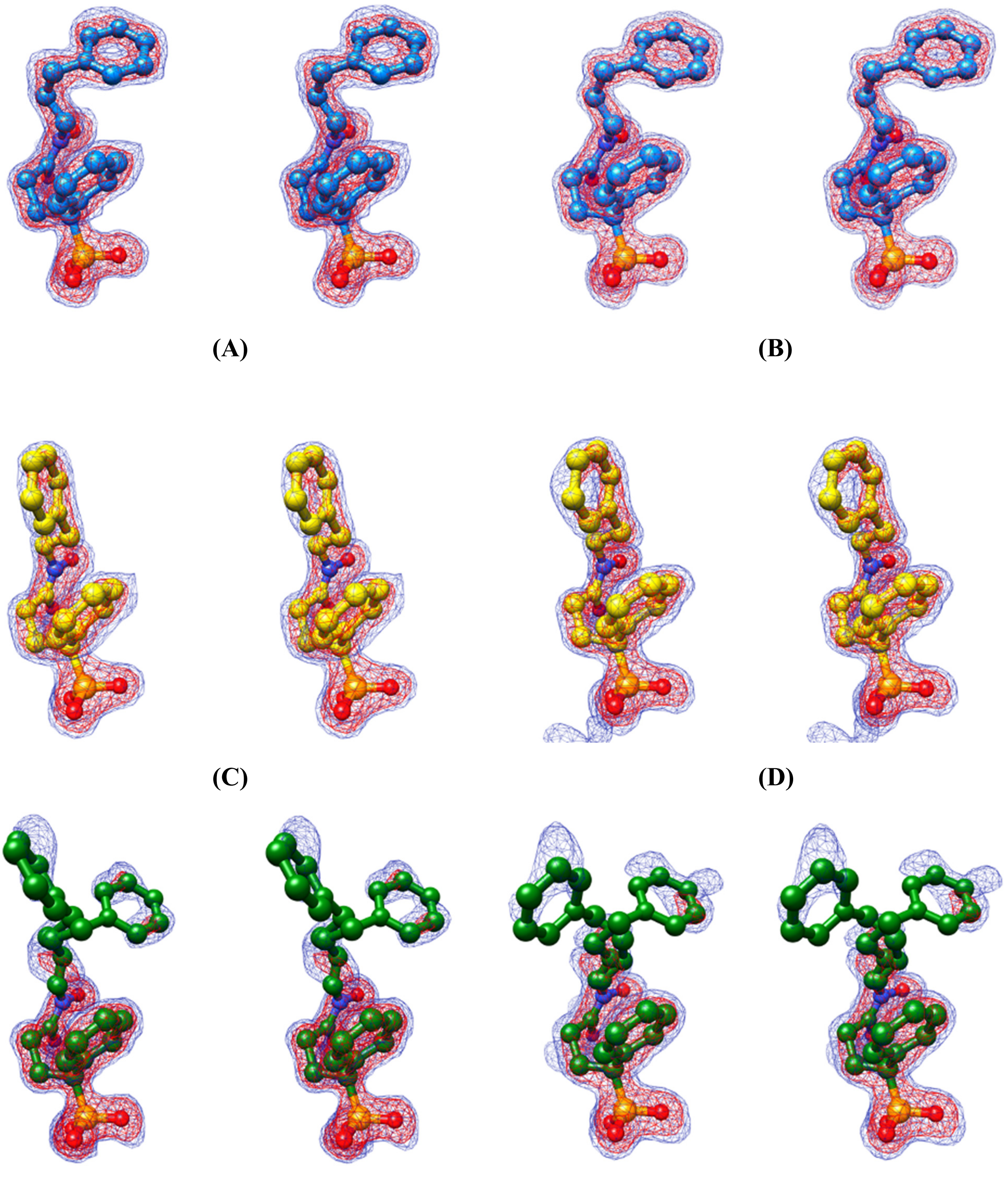

Figure S1. Stereo diagrams showing the |*F*o| - |*F*c| omit maps of the bound inhibitors in the active site of subunit B of *Pf*DXR. To eliminate any potential model bias, the structures were refined in the absence of the inhibitor molecules prior to map calculation. Subsequently, the amplitude |*F*c| and the phase angle derived from the partial structure were employed to calculate the |*F*o| - |*F*c| omit map. The contour levels are 2.0 σ (blue) and 4.0 σ (red). The inhibitor molecules are depicted as ball-and-stick models and colored in accordance with Fig. 2. (**A**) MAMK89-ternary complex at 1.50 Å resolution. (**B**) MAMK89-quaternary complex at 1.39 Å resolution. (**C**) MAMK150-ternary complex at 1.79 Å resolution. (**D**) MAMK150-quaternary complex at 1.53 Å resolution. (**E**) MAMK218-ternary complex at 1.33 Å resolution. (**F**) MAMK218-quaternary complex at 1.55 Å resolution.

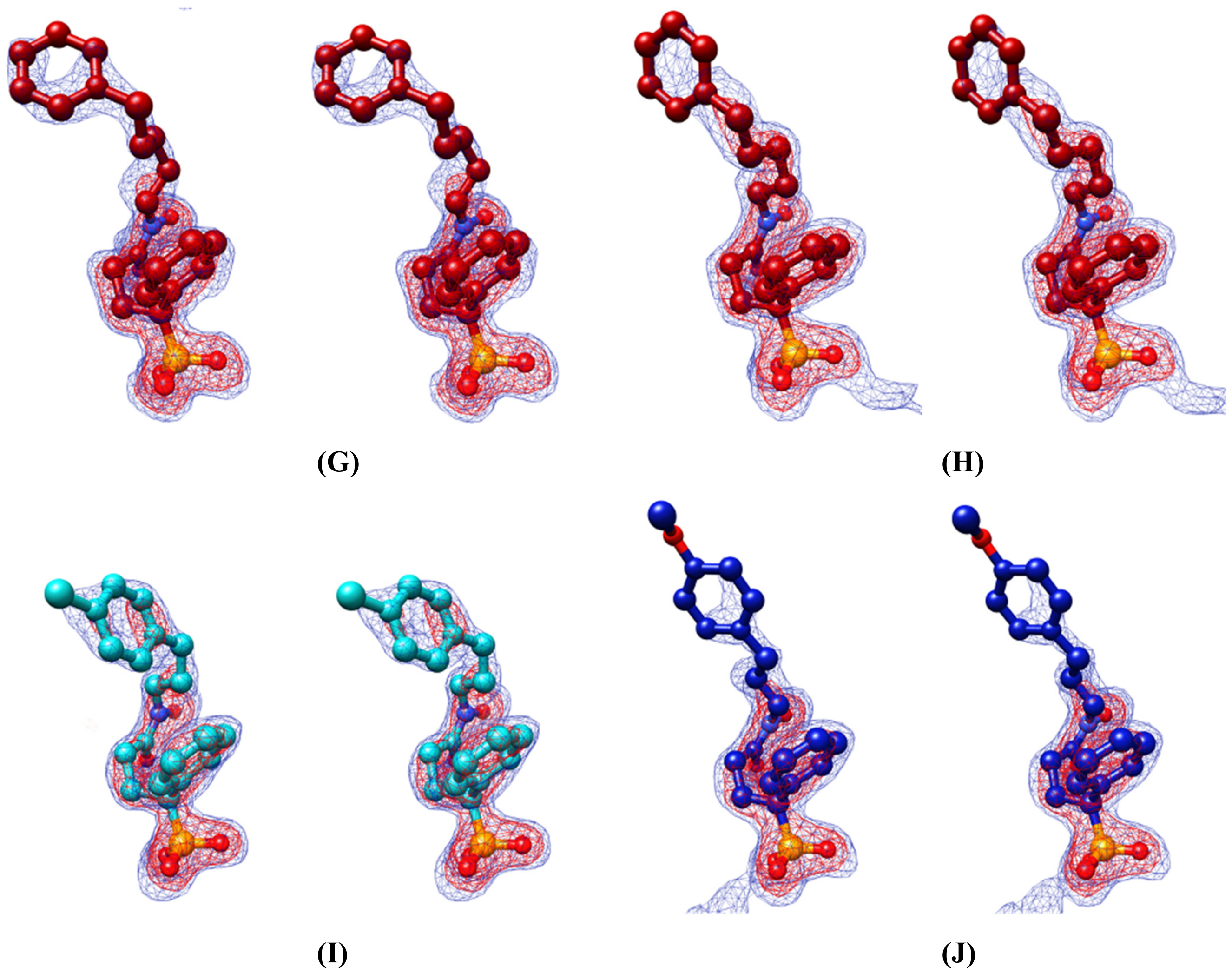

Figure S1. (continued) Stereo diagrams showing the |*F*o| - |*F*c| omit maps of the bound inhibitors in the active site of subunit B of *Pf*DXR. To eliminate any potential model bias, the structures were refined in the absence of the inhibitor molecules prior to map calculation. Subsequently, the amplitude |*F*c| and the phase angle derived from the partial structure were employed to calculate the |*F*o| - |*F*c| omit map. The contour levels are 2.0 σ (blue) and 4.0 σ (red). The inhibitor molecules are depicted as ball-and-stick models and colored in accordance with Fig. 2. (**G**) MAMK251-ternary complex at 1.47 Å resolution. (**H**) MAMK251-quaternary complex at 1.77 Å resolution. (**I**) MAMK433-quaternary complex at 1.65 Å resolution. (**J**) MAMK431-quaternary complex at 1.84 Å resolution.

Table S1. Data collection statistics for the ternary (enzyme - Mn^{2+} - inhibitor) and quaternary (enzyme - Mn^{2+} - NADPH - inhibitor) complexes of *Pf*DXR

**Data set**	**MAMK150 ternary**	**MAMK150 quaternary**	**MAMK89 ternary**	**MAMK89 quaternary**	**MAMK218 ternary**	**MAMK218 quaternary**	**MAMK251 ternary**	**MAMK251 quaternary**	**MAMK433 quaternary**	**MAMK431 quaternary**
Linker of the *N*-substituent of the bound inhibitor	ethyl	ethyl	propyl	propyl	butyl	butyl	pentyl	pentyl	propyl	propyl
**PDB ID**	**9JZ7**	**9JZ8**	**9JOA**	**9JOB**	**9JZ9**	**9JZA**	**9JZB**	**9JZC**	**9JZD**	**9JZE**
Beamline	SPring-8 BL44XU	Photon Factory BL5A	Photon Factory BL5A	Photon Factory BL17A	SPring-8 BL44XU	SPring-8 BL44XU	Photon Factory BL5A	Photon Factory BL5A	Photon Factory BL1A	Photon Factory BL1A
Wavelength (Å)	0.90000	1.00000	1.00000	0.98000	0.90000	0.90000	1.00000	1.00000	1.01200	1.01200
Detector	Dectris Eiger X 16M	Dectris Pilatus3 S 6m	Dectris Pilatus3 S 6m	Dectris Eiger X 16M	Dectris Eiger X 16M	Dectris Eiger X 16M	Dectris Pilatus3 S 6m	Dectris Pilatus3 S 6m	Dectris Eiger X 4M	Dectris Eiger X 4M
Space group	$P2_1$	$P2_1$	$P2_1$	$P2_1$	$P2_1$	$P2_1$	$P2_1$	$P2_1$	$P2_1$	$P2_1$
Cell dimensions										
a (Å)	51.749	51.608	51.637	51.518	51.934	51.536	51.516	51.464	51.596	51.564
b (Å)	77.580	77.679	77.022	78.201	77.572	77.510	76.395	77.520	76.699	77.267
c (Å)	111.575	111.062	111.275	111.144	111.659	111.271	110.727	111.475	110.166	111.427
α (°)	90	90	90	90	90	90	90	90	90	90
β (°)	92.602	92.755	92.669	92.635	93.202	92.378	92.461	92.194	92.768	92.456
γ (°)	90	90	90	90	90	90	90	90	90	90
No. of molecules per asymmetric unit	2	2	2	2	2	2	2	2	2	2
Mosaic spread (°)	0.213	0.184	0.325	0.091	0.114	0.384	0.399	0.101	0.306	0.115
Resolution (Å) (outer shell)	40.65 - 1.79 (1.82 - 1.79)	55.47 - 1.53 (1.56 - 1.53)	63.31 - 1.50 (1.53 - 1.50)	63.93 - 1.39 (1.41 - 1.39)	46.04 - 1.33 (1.35 - 1.33)	42.89 - 1.55 (1.58 - 1.55)	55.31 - 1.47 (1.50 - 1.47)	40.43 - 1.77 (1.80 - 1.77)	62.92 - 1.65 (1.68 – 1.65)	63.48 – 1.84 (1.87 – 1.84)
No. of observed reflections	223,648 (10,307)	415,780 (20,716)	438,740 (19,451)	1,032,878 (18,609)	600,735 (26,708)	391,442 (10,191)	452,830 (22,345)	278,078 (12,968)	403,435 (19,001)	303,562 (14,444)
No. of unique reflections	82,163 (3,917)	131,645 (6,510)	134,944 (6,568)	164,006 (4,569)	201,013 (9,331)	123,365 (4,844)	143,344 (6,912)	85,099 (4,154)	101,361 (4,800)	75,928 (3,610)
Completeness (%)	98.6 (95.3)	99.7 (98.3)	97.0 (94.4)	92.7 (52.3)	98.8 (92.2)	97.3 (76.2)	98.4 (95.5)	99.7 (98.0)	98.2 (93.6)	99.8 (96.7)
Redundancy	2.7 (2.6)	3.2 (3.2)	3.3 (3.0)	6.3 (4.1)	3.0 (2.9)	3.2 (2.1)	3.2 (3.2)	3.3 (3.1)	4.0 (4.0)	4.0 (4.0)
$I / \sigma(I)$	8.4 (1.9)	8.2 (1.9)	11.6 (1.0)	10.9 (1.2)	8.0 (1.0)	7.2 (1.0)	8.6 (1.0)	9.5 (1.1)	5.3 (1.0)	9.1 (1.6)
CC_{half}	0.992 (0.525)	0.996 (0.604)	0.984 (0.528)	0.998 (0.582)	0.995 (0.556)	0.948 (0.656)	0.960 (0.579)	0.997 (0.526)	0.977 (0.534)	0.994 (0.603)
R_{merge} (*I*)	0.075 (0.608)	0.068 (0.740)	0.057 (0.657)	0.066 (0.970)	0.064 (0.765)	0.074 (0.528)	0.065 (0.660)	0.070 (0.938)	0.130 (0.906)	0.091 (0.990)
R_{meas} (*I*)	0.093 (0.756)	0.082 (0.887)	0.070 (0.806)	0.072 (1.116)	0.078 (0.932)	0.088 (0.694)	0.079 (0.793)	0.084 (1.133)	0.150 (1.051)	0.105 (1.146)
R_{pim} (*I*)	0.055 (0.444)	0.045 (0.485)	0.046 (0.560)	0.028 (0.537)	0.043 (0.524)	0.048 (0.443)	0.045 (0.434)	0.046 (0.628)	0.075 (0.525)	0.052 (0.570)
Wilson *B*-factor ($Å^2$)	17.590	15.650	14.600	17.140	13.670	18.830	14.360	22.080	14.470	20.610

Table S2. Refinement statistics for the ternary (enzyme - Mn^{2+} - inhibitor) and quaternary (enzyme - Mn^{2+} - NADPH - inhibitor) complexes of *Pf*DXR

**Data set**	**MAMK150 ternary**	**MAMK150 quaternary**	**MAMK89 ternary**	**MAMK89 quaternary**	**MAMK218 ternary**	**MAMK218 quaternary**	**MAMK251 ternary**	**MAMK251 quaternary**	**MAMK433 quaternary**	**MAMK431 quaternary**
Linker of the *N*-substituent of the bound inhibitor	ethyl	ethyl	propyl	propyl	butyl	butyl	pentyl	pentyl	propyl	propyl
**PDB ID**	**9JZ7**	**9JZ8**	**9JOA**	**9JOB**	**9JZ9**	**9JZA**	**9JZB**	**9JZC**	**9JZD**	**9JZE**
Resolution (Å) (outer shell)	39.67-1.79 (1.835 - 1.79)	55.47-1.53 (1.570 - 1.53)	63.31 - 1.50 (1.539 - 1.50)	63.93 - 1.39 (1.425 - 1.39)	46.04 - 1.33 (1.362 - 1.33)	42.89 - 1.55 (1.590 - 1.55)	55.31 - 1.47 (1.508 - 1.47)	40.43 - 1.77 (1.816 - 1.77)	62.92 - 1.65 (1.692 – 1.65)	63.48 – 1.84 (1.886 – 1.84)
Completeness (%)	98.52	99.68	96.97	92.62	98.68	97.26	98.40	99.60	98.04	99.73
No. of reflections										
Working set	77,767	124,852	128,254	155,538	190,744	116,924	135,935	80,575	96,139	72,038
Test set	4,314	6,728	6,686	8,391	10,106	6,391	7,341	4,452	5,080	3,800
R-factor	0.162	0.183	0.171	0.165	0.170	0.189	0.195	0.169	0.179	0.167
Free *R*-factor	0.194	0.204	0.201	0.189	0.191	0.211	0.219	0.203	0.207	0.200
No. of protein atoms (average *B*-factors ($Å^2$))	6,570 (27.08)	6,586 (23.33)	6,570 (23.89)	6,570 (25.31)	6,562 (22.31)	6,488 (28.12)	6,530 (23.55)	6,518 (34.40)	6,518 (24.37)	6,490 (31.94)
No. of Mn^{2+} ions (ave. *B*)	2 (15.61)	2 (12.53)	2 (11.68)	2 (13.84)	2 (11.45)	2 (15.79)	2 (11.85)	2 (19.98)	2 (11.98)	2 (16.97)
No. of NADPH atoms (ave. *B*)	-	96 (21.94)	-	96 (22.27)	-	96 (27.83)	-	96 (38.15)	96 (32.54)	96 (44.77)
No. of inhibitor atoms (ave. *B*)	50 (20.90)	50 (16.70)	52 (15.33)	52 (16.48)	74 (21.57)	74 (23.44)	56 (24.20)	56 (33.81)	54 (20.75)	56 (36.89)
No. of glycerol atoms (ave. *B*)	12 (29.73)	-	12 (17.73)	-	12 (28.26)	-	12 (22.38)	12 (29.85)	-	-
No. of Ca^{2+} ions (ave. *B*)	3 (23.83)	3 (21.297)	3 (21.39)	3 (22.347)	3 (19.95)	3 (25.22)	3 (22.90)	3 (28.88)	3 (22.00)	3 (27.17)
No. of water atoms (ave. *B*)	433 (33.58)	518 (29.41)	582 (32.14)	659 (33.11)	672 (30.86)	415 (32.46)	436 (28.47)	421 (37.52)	528 (29.38)	423 (34.51)
Ramachandran plot statistics										
favored (%)	97.9	97.4	97.2	96.9	97.5	97.1	97.4	97.6	97.3	97.1
allowed (%)	2.0	2.4	2.8	3.1	2.2	2.7	2.5	2.4	2.6	2.9
outlier (%)	0.1	0.1	0	0	0.2	0.1	0.1	0	0.1	0
Root-mean-square deviations										
bonds (Å)	0.009	0.009	0.010	0.012	0.012	0.011	0.010	0.008	0.009	0.009
angles (°)	1.751	1.742	1.859	2.042	1.935	1.947	1.793	1.694	1.745	1.766

Table S3. Summary of enzyme-inhibitor interactions observed in the crystal structures of *Pf*DXR.
The color coding of the column backgrounds is as follows: green signifies vdW interactions, light blue indicates hydrogen bonding, orange denotes CH-π interactions, and pink designates metal coordination interactions.

Inhibitor	MAMK150	MAMK89	MAMK218	MAMK218	MAMK251	MAMK433	MAMK431
Conformation	curved	bent	bent	curved	curved	curved	curved
Ser88 (side chain)					*N*-Ph		*N*-4-MOPh
Asn92 (side chain)					*N*-Ph		*N*-4-MOPh
Ser232 (side chain)	*N*-Et	*N*-Pr	*N*-Bu	*N*-Bu	*N*-Pe	*N*-Pr	*N*-Pr
		N-Ph	*N*-Ph				
Ser232 N	O2 (RHA)	O2 (RHA)	O2 (RHA)	O2 (RHA)	O2 (RHA)	O2 (RHA)	O2 (RHA)
Ser232 Oγ	O2 (RHA)	O2 (RHA)	O2 (RHA)	O2 (RHA)	O2 (RHA)	O2 (RHA)	O2 (RHA)
Ser269 Oγ	O3 (Phos)	O3 (Phos)	O3 (Phos)	O3 (Phos)	O3 (Phos)	O3 (Phos)	O3 (Phos)
Ser270 N	O3 (Phos)	O3 (Phos)	O3 (Phos)	O3 (Phos)	O3 (Phos)	O3 (Phos)	O3 (Phos)
Ser270 Oγ	O5 (Phos)	O5 (Phos)	O5 (Phos)	O5 (Phos)	O5 (Phos)	O5 (Phos)	O5 (Phos)
Ser270 (side chain)	α-Ph	α-Ph	α-Ph	α-Ph	α-Ph	α-Ph	α-Ph
Lys295 (side chain)		α-Ph	*	*		*	*
Lys295 (peptide bond)		α-Ph	*	*		*	*
Trp296 (side chain)	α-Ph	*N*-Pr	*N*-Bu	α-Ph	α-Ph	α-Ph	*
	N-Ph			*N*-Ph	*N*-Ph	*N*-*p*-Tol	
					N-Pe		
Trp296 (peptide bond)		α-Ph			*N*-Ph	*N*-*p*-Tol	*
Lys297 (peptide bond)					*N*-Ph	*N*-*p*-Tol	
Met298 (side chain)	Et-link	Et-link	Et-link	Et-link	Et-link	Et-link	Et-link
	N-Et			*N*-Bu	*N*-Pe	*N*-Pr	*N*-Pr
	N-Ph			*N*-Ph	*N*-Ph	*N*-*p*-Tol	*N*-4-MOPh
Ile302 (side chain)	Et-link	Et-link	Et-link	Et-link	Et-link	Et-link	Et-link
Ser306 Oγ	O5 (Phos)	O5 (Phos)	O5 (Phos)	O5 (Phos)	O5 (Phos)	O5 (Phos)	O5 (Phos)
Asn311 Nδ	O1 (RHA)	O1 (RHA)	O1 (RHA)	O1 (RHA)	O1 (RHA)	O1 (RHA)	O1 (RHA)
	O4 (Phos)	O4 (Phos)	O4 (Phos)	O4 (Phos)	O4 (Phos)	O4 (Phos)	O4 (Phos)
Lys312 Nζ	O3 (Phos)	O3 (Phos)	O3 (Phos)	O3 (Phos)	O3 (Phos)	O3 (Phos)	O3 (Phos)
Cys338 (side chain)	α-Ph	α-Ph	α-Ph	α-Ph	α-Ph	α-Ph	α-Ph
His341 (side chain)		*N*-Ph	*N*-Ph				
		N-Ph	*N*-Ph				
Pro358 (side chain)	*N*-Ph	*N*-Ph	*N*-Ph	*N*-Ph	*N*-Pe	*N*-*p*-Tol	*N*-4-MOPh
						N-Pr	*N*-Pr
Pro358 (peptide bond)	*N*-Ph	*N*-Ph	*N*-Ph	*N*-Ph	*N*-Ph	*N*-*p*-Tol	*N*-4-MOPh
					N-Pe		
Asp359 (peptide bond)	*N*-Ph	*N*-Ph	*N*-Ph	*N*-Ph	*N*-Ph	*N*-*p*-Tol	*N*-4-MOPh
					N-Pe		
Met360 (side chain)	*N*-Et	*N*-Pr	*N*-Bu	*N*-Bu	*N*-Pe	*N*-Pr	*N*-Pr
	N-Ph	*N*-Ph	*N*-Ph	*N*-Ph	*N*-Ph	*N*-*p*-Tol	*N*-4-MOPh
Met360 (peptide bond)	*N*-Ph	*N*-Ph	*N*-Ph	*N*-Ph	*N*-Ph	*N*-*p*-Tol	*N*-4-MOPh
					N-Pe		
Pro363 (side chain)		*N*-Ph	*N*-Ph				
Mn	O1 (RHA)	O1 (RHA)	O1 (RHA)	O1 (RHA)	O1 (RHA)	O1 (RHA)	O1 (RHA)
	O2 (RHA)	O2 (RHA)	O2 (RHA)	O2 (RHA)	O2 (RHA)	O2 (RHA)	O2 (RHA)

RHA: reverse hydroxamate, Phos: phosphonate, Et-link: ethyl linker between phosphonate and hydroxamate
α-Ph: α-substituted phenyl group
N-Et, *N*-Pr, *N*-Bu, or *N*-Pe: Ethyl, Propyl, Butyl, or Pentyl linker of *N*-phenylalkyl substituent
N-Ph, *N*-*p*-Tol, or *N*-4-MOPh: Phenyl, *para*-tolyl, or 4-methoxy phenyl moiety of *N*-phenylalkyl substituent
*disordered
